# Engineering Alfalfa to Produce 2-*O*-Caffeoyl-L-Malate (Phaselic Acid) for Preventing Post-harvest Protein Loss via Oxidation by Polyphenol Oxidase

**DOI:** 10.3389/fpls.2020.610399

**Published:** 2021-01-13

**Authors:** Michael L. Sullivan, Heather A. Green, Julian C. Verdonk

**Affiliations:** US Dairy Forage Research Center, Agricultural Research Service, USDA, Madison, WI, United States

**Keywords:** BAHD acyltransferase, forage, hydroxycinnamoyl-CoA hydroxycinnamoyl transferase, protein protection, specialized metabolism

## Abstract

Many plants accumulate high levels of hydroxycinnamoyl esters and amides in their tissues, presumably to protect against biotic and abiotic stress. Red clover (*Trifolium pretense*) leaves accumulate high levels [5–15 mmol/kg fresh weight (FW)] of caffeic acid derivatives, including phaselic acid (2-*O*-caffeoyl-L-malate). Oxidation of caffeoyl-malate by an endogenous polyphenol oxidase (PPO) has been shown to help preserve forage protein after harvest and during storage as silage, which should improve N use efficiency in dairy and other ruminant production systems. The widely grown forage alfalfa lacks both PPO and PPO substrates and experiences substantial loss of protein following harvest. We previously identified a hydroxycinnamoyl-coenzyme A (CoA):malate hydroxycinnamoyl transferase (HMT, previously called HCT2) responsible for phaselic accumulation in red clover. With the goal of producing PPO-oxidizable compounds in alfalfa to help preserve forage protein, we expressed red clover HMT in alfalfa. Leaves of these alfalfa accumulated mainly *p*-coumaroyl- and feruloyl-malate (up to 1.26 and 0.25 mmol/kg FW, respectively). Leaves of HMT-expressing alfalfa supertransformed with an RNA interference (RNAi) construct to silence endogenous caffeoyl-CoA acid *O*-methyltransferase (CCOMT) accumulated high levels of caffeoyl-malate, as well as the *p*-coumaroyl and feruloyl esters (up to 2.16, 2.08, and 3.13 mmol/kg FW, respectively). Even higher levels of caffeoyl- and *p*-coumaroyl-malate were seen in stems (up to 8.37 and 3.15 mmol/kg FW, respectively). This level of caffeoyl-malate accumulation was sufficient to inhibit proteolysis in a PPO-dependent manner in *in vitro* experiments, indicating that the PPO system of post-harvest protein protection can be successfully adapted to alfalfa.

## Introduction

Hydroxycinnamoyl esters and amides can serve a variety of roles in plants. One well characterized example is *p*-coumaroyl-shikimate, which is essentially a biosynthetic intermediate between *p*-coumaroyl-Coenzyme A (CoA) and caffeoyl-CoA by being the substrate for coumaroyl 3′-hydroxylase (C3′H) (Schoch et al., 2006). C3′H is a cytochrome P450 that hydroxylates the *p*-coumaroyl moiety of the ester to the caffeoyl moiety, which can subsequently be transformed to other hydroxycinnamates such as ferulic and sinapic acid. These biochemical transformations are important in the biosynthesis of monolignol precursors for lignin formation and volatile phenylpropanoids/benzenoids (Dudareva et al., 2013; Dixon and Barros, 2019). In other cases, hydroxycinnamoyl derivatives accumulate to relatively high levels where they likely serve to protect the plant against biotic and abiotic stresses (Niggeweg et al., 2004). For example, arabidopsis and other brassica species accumulate sinapoyl-malate in their leaves (Lehfeldt et al., 2000), presumably as protection against UV radiation (Jin et al., 2000). Coffee beans accumulate large amounts of chlorogenic acid (caffeoyl-quinate) (Lallemand et al., 2012). Tomato, potato, and other solanaceous species also accumulate chlorogenic acid and other hydroxycinnamoyl-quinate esters (Niggeweg et al., 2004). In the case of the caffeoyl derivatives, these can be the substrate for endogenous chloroplast-localized polyphenol oxidases (PPOs). Upon tissue breakdown and release of PPO, oxidation of these caffeic acid derivatives to the corresponding quinones, and the subsequent secondary reactions of the quinones can lead to the familiar browning reaction common in injured or post-harvest plant materials (Sullivan et al., 2004; Sullivan and Hatfield, 2006; Sullivan and Foster, 2013). These reactions appear to also have a role in protecting plants against herbivory and pathogens (Thipyapong et al., 2004).

The forage crop red clover (*Trifolium pretense* L.) contains high levels of PPO activity (Sullivan et al., 2004) and accumulates two caffeoyl-derivatives that are oxidizable by PPO: clovamide (*N*-caffeoyl-3,4-dihydroxyphenylalanine) and phaselic acid (2-*O*-caffeoyl-L-malate, hereafter referred to as caffeoyl-malate whereas related compounds will be referred to as *p*-coumaroyl-malate, feruloyl-malate, or hydroxycinnamoyl-malate for these compounds in general) (Sullivan and Zeller, 2012). In red clover leaves, caffeoyl-malate can accumulate to 4–15 mmol/kg fresh weight (FW) (Sullivan and Zeller, 2012, and references therein) where, as described above, it presumably helps defend the plant against abiotic and biotic stress. Post-harvest, as plant tissues break down, the action of PPO on these caffeoyl derivatives, while leading to the familiar browning, also prevents degradation of protein to amino acids during storage as silage (Sullivan and Hatfield, 2006). This proteolytic inhibition is presumably due to quinones reacting with nucleophilic sites on proteins, either inactivating endogenous plant proteases themselves, making proteins poor substrates for endogenous proteases, or both. Keeping plant protein intact (i.e., “true protein”) is important because ruminant animals, such as dairy cows, poorly use degraded protein, leading to increased cost to producers because they need to supplement diets with true protein. High levels of degraded protein in ruminant diets also leads to excess nitrogen being released into the environment since degraded protein tends to be excreted by the animal rather than incorporated into milk or meat (Rotz et al., 1999). Previously, we showed red clover’s natural system of protein protection could likely be transferred to the widely used perennial forage alfalfa, which lacks both endogenous foliar PPO activity, but also PPO-oxidizable substrates such as caffeoyl-malate or other caffeic acid derivatives (Sullivan et al., 2004; Sullivan and Hatfield, 2006; Sullivan et al., 2008).

**FIGURE 1:**
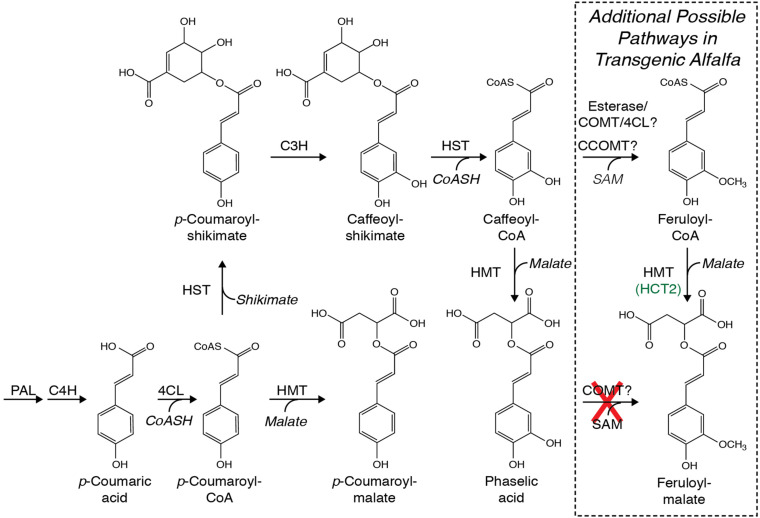
Possible pathways for biosynthesis of caffeoyl-malate (phaselic acid) and *p*-coumaroyl-malate that include phenylalanine ammonia lyase (PAL), cinnamate 4-hydroxylase (C4H), 4-coumarate:CoA ligase (4CL), hydroxycinnamoyl-CoA:shikimate hydroxycinnamoyl transferase (HST), hydroxycinnamoyl-CoA:malate hydroxycinnamoyl transferase (HMT), and *p*-coumarate 3′-hydroxylase (C3H). No enzymatic activity in red clover has been identified that can directly hydroxylate *p*-coumaroyl-malate to caffeoyl-malate (Sullivan and Zarnowski, 2010). Additional pathways whereby feruloyl-malate might be made in alfalfa are boxed and include the enzymes caffeoyl-CoA *O*-methyltransferase (CCOMT) and caffeic acid *O*-methyltransferase (COMT), possibly in conjunction with esterases and 4CL. As detailed in the text, COMT is incapable of direct methylation of caffeoyl-malate as indicated with a red “X”.

Although in arabidopsis and some other Brassicaceae, hydroxycinnamoyl-malate esters are synthesized via the action of a hydroxycinnamoyl-glucose hydroxycinnamoyl transferase (Lehfeldt et al., 2000), in common bean (*Phaseolus vulgaris* L.) (Sullivan, 2017) and red clover (Sullivan, 2009), we identified BAHD family (D’Auria, 2006) hydroxycinnamoyl-CoA:malate hydroxycinnamoyl transferases capable of transferring hydroxycinnamic acids from a CoA thiolester to malic acid (Figure 1). In red clover, the transferase, HMT (previously called HCT2), appears to be responsible for caffeoyl-malate accumulation (Sullivan and Zarnowski, 2011). Similar hydroxycinnamoyl-CoA transferases are responsible for biosynthesis of chlorogenic acid and related compounds in coffee (Lallemand et al., 2012) and solanaceous species like tomato (Niggeweg et al., 2004; Cle et al., 2008), and of *p*-coumaroyl-shikimate in several species including arabidopsis, alfalfa, and red clover (Hoffmann et al., 2003, 2004; Shadle et al., 2007; Sullivan, 2009). Here, we describe using red clover HMT to make a biosynthetic pathway in alfalfa to produce caffeoyl-malate as a PPO substrate with the goal of recreating red clover’s PPO/o-diphenol system of post-harvest protein protection in this important forage crop.

## Materials and Methods

### Reagents

All purchased reagents were of molecular biology or higher grade. The standard 2-*O*-(*p-*coumaroyl)-L-malate was prepared by the method described by Hemmerle et al. (1997) and characterized as previously detailed (Sullivan and Zarnowski, 2011). 2-*O*-caffeoyl-L-malate was synthesized and characterized as reported (Zeller, 2012). 2-*O*-feruloyl-L-malate was prepared enzymatically using HMT protein as previously described (Sullivan and Zarnowski, 2011).

Caffeoyl-, *p*- coumaroyl-, and feruloyl-CoA thiolesters were prepared using recombinant arabidopsis (*Arabidopsis thaliana*) 4CL1 protein (Lee et al., 1995) produced in *Escherichia coli* using the pET30 expression vector (Novagen MilliporeSigma, Burlington, MA, United States) as previously detailed (Sullivan, 2009). Concentrations of the thiolesters were determined spectrophotometrically using extinction coefficients of 21, 18, and 19 mM^–1^ cm^–1^ for *p*-coumaroyl- (λ_max_ = 333 nm), caffeoyl- (λ_max_ = 346 nm), and feruloyl-CoA (λ_max_ = 346 nm), respectively (Stoeckigt and Zenk, 1975).

### Plant Materials

All work described here used a highly regenerable alfalfa (*Medicago sativa*) clone (Regen SY27) (Bingham, 1991; Samac and Austin-Phillips, 2006). Both wild type and transgenic plants (described below) were propagated by stem cuttings. Plants were maintained in a greenhouse year-round and fertilized weekly (Peter’s soluble 20-20-20; Scotts, Marysville, OH, United States). Supplemental lighting (13 h/d) was used during all but summer months. For experiments detailed here, plant tissue was typically harvested mid to late morning. For analysis of mRNA levels and hydroxycinnamoyl ester content leaves were harvested, frozen in liquid nitrogen, and stored at −80°C until needed.

### Nucleic Acid Methodologies

Total RNA was prepared from plant tissues using the RNeasy Plant Mini Kit (Qiagen, Valencia, CA, United States). Oligo dT-primed cDNA was prepared using Superscript III reverse transcriptase according to the manufacturer’s protocol (Invitrogen, Carlsbad, CA, United States) from DNase I-treated total RNA. Plasmid DNA was prepared using the QIAprep Spin Miniprep Kit (Qiagen). Alfalfa DNA for PCR screening was prepared from freshly harvested leaves by extraction with Tris/EDTA/SDS followed by precipitation with ammonium acetate and isopropyl alcohol. DNA sequence was determined by Sanger cycle sequencing using Big Dye v3.1 (Applied Biosystems, Foster City, CA, United States) and run on ABI 3730xl DNA Analyzers by the University of Wisconsin Biotechnology Center (Madison, WI, United States). Sequence analyses were carried out using the Lasergene Version 8 or higher (DNAStar, Madison, WI, United States), and BLAST programs using the National Center for Biotechnology Information (NCBI)^1^ web sites.

### Cloning and Plasmid Construction

For all cloning, standard molecular biology techniques were used (Sambrook et al., 1989; Ausubel et al., 1998). When fragments for cloning were generated via PCR, a high-fidelity thermostable enzyme, Phusion DNA polymerase (New England Biolabs, Ipswich, MA, United States), was used according to manufacturer’s suggested conditions and the cloned insert was sequenced to ensure that no mutations were introduced.

For expression of red clover HMT in transgenic alfalfa, a PCR primer pair (ms536 and ms537, Table 1) was designed to introduce *Xba*I and *Bam*HI restriction endonuclease sites on either side, respectively, of the red clover HMT coding region (Sullivan, 2009). The forward primer contained the proposed dicot consensus sequence AAACA (Joshi et al., 1997) immediately upstream of the initiating Met codon. A cloned red clover HMT cDNA (GenBank EU861219) served as template for PCR (Sullivan, 2009). The resulting HMT PCR product was digested with *Xba*I and *Bam*HI and cloned into the plant transformation vector pILTAB357 (also digested with *Xba*I and *Bam*HI) (Verdaguer et al., 1996) such that the HMT open reading frame was between the vector’s cassava vein mosaic virus (CsVMV) promoter and the NOS (nopaline synthase) terminator.

**TABLE 1:** Oligo nucleotide primers used in this study.

Designation	Sequence^a^ (5′–3′)
ms74	GCTATGACTGGGCACAACAGAC
ms75	CGTCAAGAAGGCGATAGAAGG
ms507	GCGGGTTCGGCCCATTCGGACC
ms508	GCCCTCGGACGAGTGCTGGGG
ms524	AGTTGGGAAATTGGGTTCGAAATCG
ms525	TCATTAAAGCAGGACTCTAGAGGATC
ms536	GTCTAGA*AAACA*ATGGTTACCATTAAAAATTCTTACAC
ms537	GGGATCCTCATATATCCTCATAGAAGTACTTTTTG
ms638	GGGGGATCCATGAAAGAGTTGAGAGAGGTCAC
ms639	GGGCTCGAGCCATGAAAGAGTTGAGAGAGGTCAC
ms640	GGGATCGATACAGCCAAAGCCTTGTTAAGCTCC
ms641	GGGGGTACCACAGCCAAAGCCTTGTTAAGCTCC
ms646	GCATATGGGTTCAACAGGTGAAACTCAAATAAC
ms647	GCTCGAGTTAAACCTTCTTAAGAAACTCCATGATG
ms650	CGGCCAGACAATGGAAAAGTGA
ms651	TGAATCTGGAGCCACTGGAAGT
ms666	AGACATCAAGAAGTTGGTCACAAGA
ms667	GGGAAGACACTGGTCTCTAGAATATACTG
ms786	ACCATCAATGATCGGAATGG
ms787	ATGATAGAGTTGTAGGTGGTCTCGT

*^*a*^Where appropriate, introduced restriction sites are underlined and an introduced dicot translational consensus sequence (Joshi et al., 1997) is italicized.*

To create an RNA interference (RNAi) construct to downregulate endogenous alfalfa caffeoyl-CoA *O*-methyltransferase (CCOMT), sense and antisense fragments corresponding to a 540-bp portion of an alfalfa CCOMT coding region (Genbank U20736) were generated from alfalfa leaf cDNA by PCR using the primer pairs ms638 and ms640 (to produce the sense arm) or ms639 and ms641 (to produce the antisense arm) (Table 1). Based on BLAST searches of *M. sativa* sequences available in Genbank, no obvious potential off-targets were identified. The resulting fragments were digested with either *Xho*I and *Kpn*I or *Bam*HI and *Cla*I and cloned into the sense and antisense arms, respectively, of pMLS380, a modified version of the intron-containing gene silencing vector pHANNIBAL (Wesley et al., 2001) where the 35S promoter has been replaced with the CsVMV promoter (Verdaguer et al., 1996). The silencing cassette of this pMLS380 construct was subcloned as a *Not*I fragment into pMLS298, a version of the binary vector pBIG-HYG (Becker, 1990) whose native *Not*I site was removed (cleaved, filled in, and religated) and whose polylinker was modified to have a unique *Not*I site. The final construct had the promoters for the hairpin silencing RNA and the hygromycin resistance selectable marker in a divergent orientation.

For expression of alfalfa caffeic acid *O*-methyltransferase (COMT) in *E. coli*, alfalfa leaf cDNA was used as template in PCR reactions with primers designed based on a previously cloned COMT sequence (Genbank M63853; Gowri et al., 1991) to introduce an *Nde*I site at the start codon and an *Xho*I site immediately following the stop codon of the open reading frame (Table 1, ms646 and ms647, respectively). PCR fragments from three independent PCR reactions were sequenced to ensure an error free clone whose sequence was deposited in Genbank (GU066087). The resulting PCR product was digested with *Nde*I and *Xho*I and inserted into pET42 (Novagen MilliporeSigma) digested with *Nde*I and *Xho*I, respectively. This resulted in a plasmid that would produce native COMT protein.

### Generation and Identification of Transgenic Alfalfa

Plant transformation constructs (HMT expression, CCOMT RNAi, or pMLS298 empty vector control) were introduced into *Agrobacterium tumefaciens* strain LBA4404 by standard methods (Wise et al., 2006). The resulting *A. tumefaciens* strains were used to transform a highly regenerable clone of Regen-SY27 (Bingham, 1991) (or supertransform alfalfa already transformed with the HMT transgene) as described by Samac and Austin-Phillips (2006) using 50 mg/L kanamycin or 25 mg/L hygromycin B for selection as appropriate. Plants containing transgenes were identified by PCR with GoTaq Green Master Mix (Promega Corporation, Madison, WI) using manufacturers suggested conditions and appropriate primers (Table 1) to detect the kanamycin selectable marker (ms74 and ms75), the hygromycin selectable marker (ms507 and ms508), the HMT gene (ms536 and ms537), or the antisense arm of the RNAi silencing cassette (ms524 and ms525, which anneal to the pyruvate orthophosphate dikinase (pdk) intron and OCS (octopine synthase) terminator, respectively, of the silencing cassette).

### Expression of Alfalfa COMT in *E. coli* and COMT Activity Assay

The COMT expression plasmid described above and the corresponding pET42 empty vector control were transformed into BL21(DE3)RIL Codon Plus *E. coli* (Agilent, Santa Clara, CA, United States), and the resulting *E. coli* were cultured, induced, and extracts prepared essentially as described previously (Sullivan, 2009). COMT activity was assessed by a modification of the method described by Inoue et al. (1998). *In vitro* reactions (250 μL total volume) containing 100 mM Tris (pH 7.5), 0.2 mM MgCl_2_, 10% (v/v) glycerol, 2 mM dithothreitol, 25 mM ascorbate, 0.3 mM *S*-adenosylmethione, 5 mM caffeic acid (or caffeoyl-malate), 3 μL *E. coli* extract (equivalent to 15 μL induced culture, approximately 2.5 μg COMT protein based on Coomassie staining) were incubated for up to 180 min at 30°C. Following incubations, reactions were stopped by the addition of 1/5 volume 10% formic acid, centrifuged at 17,000 × g for 5 min to remove precipitated protein, and analyzed for methylation products by liquid chromatography (LC) as described below.

### Quantitative Real-Time PCR Analysis of Gene Expression

To assess mRNA levels of endogenous CCOMT and COMT in alfalfa, cDNA was prepared from DNase I treated total RNA isolated from young fully expanded alfalfa leaves as described above. Quantitative real-time PCR was carried out using PowerUp SYBR Green PCR Master Mix (Thermo Fisher Scientific, Waltham, MA, United States) in triplicate 10 μL reactions. Each reaction contained cDNA equivalent to 12.5 ng total RNA and primers at 300 nM. Primer pairs (Table 1) were ms666/ms667 to detect alfalfa CCOMT, ms650/ms651 to detect alfalfa COMT, and ms786/ms787 to detect alfalfa actin 2 (GenBank JQ028730) as the reference gene. Real-time PCR was run in a QuantStudio 5 (Thermo Fisher Scientific) using the cycling conditions 50°C for 2 min, 95°C for 2 min, followed by 40 cycles of 95°C for 15 s, 58°C 15 for 15 s, 72°C for 1 min with CYBR as the reporter and ROX as the passive reference. Following cycling, melt curves were generated using the default settings (65–95°C over 400 s). Threshold cycle (*C*_T_) was determined using the auto function of the QuantStudio analysis software.

### Extraction of Phenolic Compounds From Plant Tissues

Tissue samples were ground in liquid nitrogen in 2 mL screw cap tubes with two 4 mm glass beads using a Mini-BeadBeater (Biospec Products, Bartlesville, OK, United States). The ground frozen tissue was extracted at room temperature with 10 mL/g FW 100 mM HCl, 50 mM ascorbic acid for 30 min with periodic mixing. Extracts were filtered through Miracloth (MilliporeSigma) or glass wool then centrifuged at 20,000 × g at room temperature. One milliliter of the resulting supernatant was applied to a 1 mL ENVI-18 solid phase extraction column (MilliporeSigma) preequilibrated with 3 × 1 mL of methanol and 3 × 1 mL 0.1% (v/v) acetic acid in water, pH adjusted to 2.5 with HCl. The column was washed with 3 × 1 mL 0.1% acetic acid (v/v) in water (pH 2.5 with HCl) and phenolic compounds eluted with 1 mL methanol.

### LC and Mass Spectrometry

Phenolic samples were analyzed by LC on a Shim-Pack XR-ODS II (C-18) 120Å column (Shimadzu Scientific Instruments North America, Columbia, MD, United States; 100 × 2.0 mm x 2.2 micron) using a two solvent system [Solvent A: deionized water with 0.1% (v/v) formic acid, Solvent B: acetonitrile] as previously described (Sullivan, 2014). Compound elution was monitored (250–500 nm) with a UV/visible photodiode array (PDA) detector. In most cases, elution was also monitored with a MS2020 mass spectrometer (MS) (Shimadzu Scientific Instruments North America) using a dual ion source (electrospray and atmospheric pressure chemical ionization) with data collection in both positive and negative ion modes. MS data was collected between 2.0 and 16.0 min of the LC run, scanning for (*m/z*) between 50 and 500 at 7,500 u/s, with detector voltage of 1.3 kV, nebulizing gas flow of 1.5 L/min, drying gas flow of 10 L/min, desolvation line and heat block temperatures of 250°C.

Compounds of interest were quantified from peak areas of PDA chromatograms (250–500 nm) using LC Solutions software (Shimadzu Scientific Instruments North America) and standard curves generated using purchased free hydroxycinnamic acids (*p*-coumaric, caffeic, and ferulic acids) as previously described (Sullivan, 2009).

### Proteolysis Assay

To prepare tissue extracts for proteolysis assays, alfalfa leaves and stems were harvested, and powdered in liquid nitrogen in a mortar and pestle. The ground tissue was extracted with 3 mL/g 0.05 M MES (2-[*N*-morpholino]ethanesulfonic acid), pH 6.5. The slurry was filtered through Miracloth and the resulting filtrate was centrifuged at 15,000 × g for 10 min at 4°C. To determine caffeoyl-malate content, a 1 mL portion of extract was acidified by adding 1/10 volume 1 N HCl and processed for phenolic content as described above. To provide a source of PPO, leaf extracts were similarly prepared from PPO-expressing alfalfa plants (Sullivan et al., 2004) or from non-transformed plants as a negative control, but in this case the post-centrifugation supernatant was desalted on spin columns packed with Sephadex G-25 (GE Healthcare, Uppsala, Sweden) equilibrated with 0.05 M MES pH 6.5 as previously described (Sullivan and Hatfield, 2006). Protein concentrations were determined using Bio-Rad Protein Assay (Bio-Rad Laboratories, Hercules, CA, United States) with bovine serum albumin as the standard.

Proteolysis assay reactions contained 2 mg/mL extract protein in 0.05 M MES, pH 6.5. The reactions consisted of 90% (v/v) extract from leaves and stems of phenolic producing plants (or their corresponding control) and 10% (v/v) leaf extract from PPO-expressing plants (or their corresponding non-transformed control). Duplicate samples of each reaction were removed at time 0 and at 24 h following incubation at 37°C, a temperature previously established for sensitive assay of amino acid release (Sullivan et al., 2004; Sullivan and Hatfield, 2006). Once removed, the samples were immediately mixed with one-half volume of 15% (w/v) trichloroacetic acid (TCA) and placed on ice for at least 30 min to precipitate proteins and peptides. TCA-insoluble material was removed by centrifugation at 20,000 × g for 5 min. Free amino acids in the resulting supernatants were measured by ninhydrin assay as previously described (Sullivan and Zeller, 2012) using glycine as the standard. Amino acid release was determined by subtracting the amino acid concentration of the initial (unincubated proteolysis reactions) from the amino acid concentrations of the reactions following incubation. Proteolysis was expressed for the PPO-containing reactions relative to the no PPO-control reactions.

### Data Analysis and Statistics

Descriptive statistics for hydroxycinnamoyl-malate content for populations of transgenic plants include range, median, and median absolute deviation (MAD), since transgenic plant data is often non-normal. A minimum of five independent transformants was analyzed (one technical replicate) for each population. Data were visualized with box and whisker plots using BoxPlotR^2^. Because of the non-normality of the hydroxycinnamoyl-malate accumulation data, non-parametric tests were used to assess whether differences in accumulation were significant (Nap et al., 1993), using directional hypotheses when appropriate. For differences in hydroxycinnamoyl-malate content between leaves of RNAi and vector control plants, the Mann-Whitney *U*-test was performed using an online tool^3^. For differences in hydroxycinnamoyl-malate ester content between leaves and stems, the Wilcoxon sign-rank test was performed on the paired data using an online tool^4^. For correlation analysis of accumulation of various hydroxycinnamoyl esters with each other and of caffeoyl-malate content with PPO mediated proteolytic inhibition, significance was tested by linear regression and Student’s *t*-test (Samuels, 1989). The analysis used proteolysis and phenolic content values from five independently prepared extracts from caffeoyl-malate-accumulating plants and the median value of three wild type plants (negative control). Real-time PCR data were analyzed by the comparative C_T_ method (Schmittgen and Livak, 2008) using the average C_T_ of three technical replicates for each sample and primer combination. ΔC_T_ was determined subtracting actin C_T_ from CCOMT C_T_ or COMT C_T_ for each sample. ΔΔC_T_ was determined by subtracting the average vector control group ΔC_T_ for CCOMT or COMT from each sample ΔC_T_ for CCOMT or COMT, respectively. Differences between treatment groups [vector control (*n* = 4 independent transformants) or RNAi accumulating caffeoyl-malate (*n* = 5 independent transformants)] were evaluated by *t*-test using Prism 8 (GraphPad Software, San Diego, CA, United States) with summary statistics presented as mean ± standard error.

## Results and Discussion

### Expression of Red Clover HMT in Alfalfa Results in Accumulation of Hydroxycinnamoyl-Malate Esters

We previously identified a hydroxycinnamoyl-CoA:malate hydroxycinnamoyl transferase (HMT, previously called HCT2) (Sullivan, 2009) from red clover capable of transferring *trans*-hydroxycinnamic acids from their CoA thiolester derivatives to L-malic acid and responsible for accumulation of hydroxycinnamoyl-malate esters, particularly caffeoyl-malate, in red clover (Figure 1; Sullivan and Zarnowski, 2011). Because PPO-oxidizable compounds like caffeoyl-malate are an important part of a natural system of post-harvest protein protection in some legume forage crops (Sullivan and Hatfield, 2006; Sullivan and Zeller, 2012; Sullivan and Foster, 2013), we sought to determine whether alfalfa, an important forage crop lacking both foliar expression of PPO and accumulation of PPO substrates, could be engineered to accumulate caffeoyl-malate.

**FIGURE 2:**
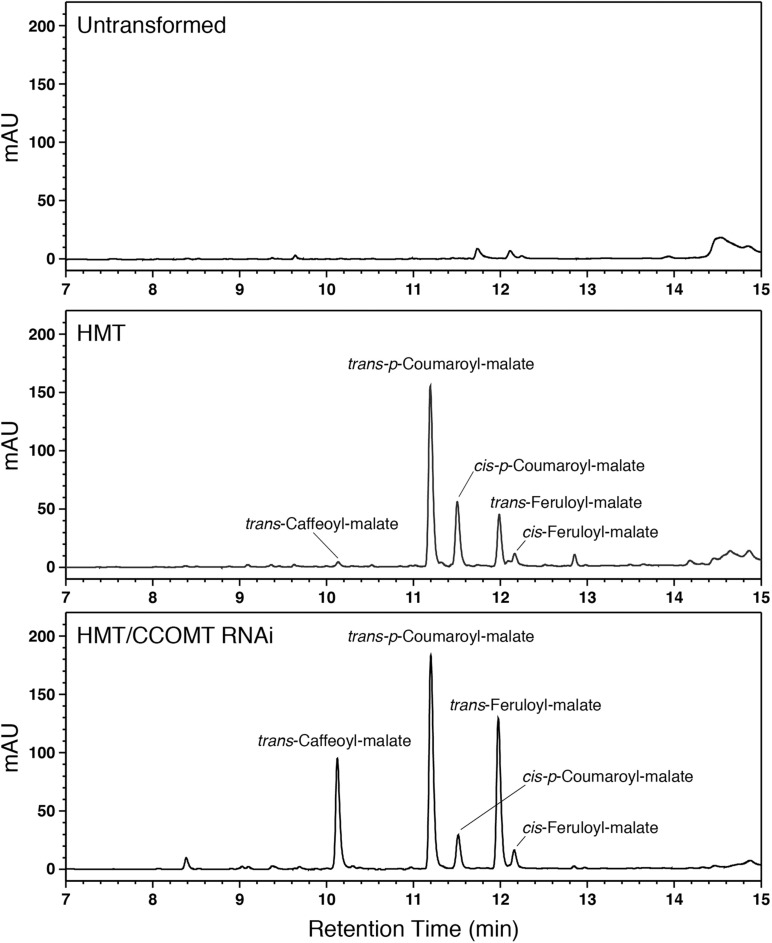
LC separation with PDA detection (310–325 nm) of alfalfa phenolics from leaves of untransformed wild type (top), HMT-expressing (middle), or HMT-expressing/CCOMT RNAi (bottom) alfalfa. Identified hydroxycinnamoyl-malate esters are indicated.

We therefore transformed alfalfa with a cDNA encoding red clover HMT under the control of the strong constitutive CsVMV promoter (Verdaguer et al., 1996). Eight independent transgenic lines containing the HMT transgene were identified by PCR. No obvious morphological or developmental phenotypes were apparent for the transgenic alfalfa relative to non-transformed alfalfa. To determine whether the HMT transgene was directing accumulation of hydroxycinnamoyl-malate esters in the alfalfa, phenolic compounds were extracted from leaves and analyzed by LC. Figure 2 shows comparison of reverse phase separation of phenolics extracted from leaves of an untransformed alfalfa plant with leaves of a typical plant transformed with red clover HMT. Several prominent peaks not present in untransformed alfalfa can be seen in the HMT-expressing plants. We identified two of these as *trans*-*p*-coumaroyl-malate and *trans*-feruloyl-malate based on them being indistinguishable from synthesized standard compound in terms of retention time (11.2 and 12.0 min, respectively), UV absorption spectrum (λ_max_ of 313 and 326 nm, respectively), and (*m/z*) (−279 and −309, respectively, in negative mode). *cis*-*p*-Coumaroyl-malate and *cis*-feruloyl-malate were also identified based on (*m/z*) and compounds of the same retention time and UV absorption spectrum formed upon UV irradiation of synthesized standards (Sullivan, 2014). The presence of *cis* versions of these compounds was not unexpected. We have previously observed *cis* versions of hydroxycinnamic acid derivatives in the leaves of both wild type plants that accumulate hydroxycinnamoyl ester compounds (e.g., red clover and perennial peanut; Sullivan, 2014) and transgenic plants expressing hydroxycinnamoyl-CoA hydroxycinnamoyl transferases (Sullivan, 2017, 2019), which are presumably in equilibrium with the *trans* versions *in vivo*. A smaller peak from the HMT alfalfa plants was identified as *trans*-caffeoyl-malate based on being indistinguishable from synthesized standard compound in terms of retention time (10.2 min), UV absorption spectrum (λ_max_ of 326 nm), and (*m/z*) (−295 in negative mode). No *cis* version was apparent, consistent with previous observations that for caffeic acid, accumulation of the *cis* isomer seems to be energetically unfavored for the free acid and at least some of its esters that have been examined (Sullivan, 2014).

**FIGURE 3:**
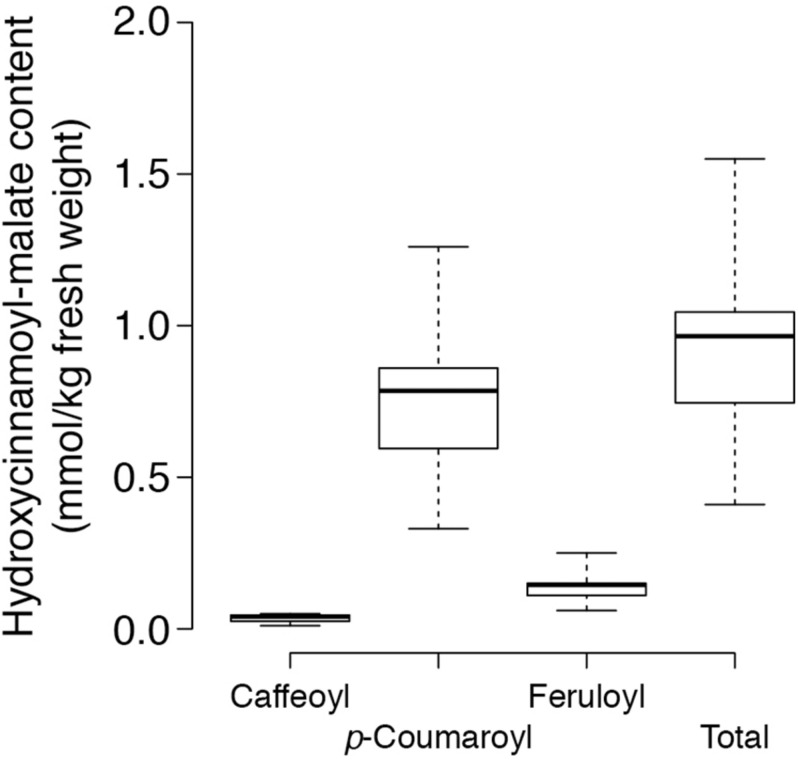
Content of various hydroxycinnamoyl-malate esters in leaves of eight independent HMT alfalfa transformants. Center lines show the medians, box limits indicate the 25th and 75th percentiles as determined by R software, whiskers extend to the minimum and maximum values.

*trans*-Hydroxycinnamoyl-malate esters accumulating in the leaves of the eight independent transgenic plants were quantified. Results are represented in a box and whiskers plot (Figure 3) with summary statistics (Table 2, see also Supplementary Data). All eight independent lines expressing red clover HMT accumulated hydroxycinnamoyl-malate esters, with total accumulation ranging from 0.41 to 1.55 mmol/kg fresh weight (0.97 ± 0.22 mmol/kg FW, median ± MAD) although these levels are lower than that typically seen for red clover (values ranging from 4 to 15 mmol/kg FW have been reported; Winters et al., 2008; Sullivan, 2009; Saviranta et al., 2010; Sullivan and Zarnowski, 2011; Sullivan and Zeller, 2012). While total *trans*-hydroxycinnamoyl-malate accumulation in the leaves varied nearly fourfold among the transgenic plants, the relative proportion of the various hydroxycinnamoyl-malate esters was relatively consistent among the independent transgenic lines, with median accumulation (as percent of total ± MAD) of 4 ± 1, 82 ± 1, and 15 ± 1 for caffeoyl-, *p*- coumaroyl-, and feruloyl-malate esters, respectively. The relatively low accumulation of caffeoyl-malate is consistent with measured kinetic parameters of red clover HMT: although caffeoyl-, *p*- coumaroyl-, and feruloyl-CoA have similar *K*_M_ values, *V*_max_ values for *p*-coumaroyl- and feruloyl-CoA are eight- and sixfold higher than for caffeoyl-CoA (Sullivan and Zarnowski, 2011). Interestingly, red clover leaves do not accumulate measurable amounts of feruloyl-malate, and accumulate predominantly caffeoyl-malate: > 90% in mature leaves, but generally lower amounts (60–70%) in young and unexpanded leaves with the balance as *p*-coumaroyl-malate (Sullivan and Zarnowski, 2011; Sullivan and Zeller, 2012). Given the apparent lack of a 3′ hydroxylating activity capable of producing caffeoyl-malate directly from *p*-coumaroyl-malate *in vivo* in red clover (Sullivan and Zarnowski, 2010), the accumulation of mostly caffeoyl-malate in red clover and mostly *p*-coumaroyl- and feruloyl-malate in HMT-expressing alfalfa could be a reflection of differences in pool sizes of the various hydroxycinnamoyl-CoA donor substrates between these two species. To our knowledge, the relative abundances of these in alfalfa and red clover leaves have not been measured. Nonetheless, since ferulic acid is derived from caffeic acid, accumulation of feruloyl-malate in alfalfa transformed with HMT suggests that the limited accumulation of caffeoyl-malate in these plants is not due exclusively, if at all, to a limitation of enzymes involved in biosynthesis of caffeoyl moieties, and that conversion of caffeoyl moieties to feruloyl moieties is contributing to the low level of caffeoyl-malate accumulation.

**TABLE 2:** Accumulation of hydroxycinnamoyl esters in leaves of eight independent HMT transformants.

	*trans*-Hydroxycinnamoyl-malate content			
	
Parameter	Caffeoyl	*p*-Coumaroyl	Feruloyl	Total
Range (mmol/kg FW)	0.01–0.05	0.33–1.26	0.06–0.25	0.41–1.55
Median ± MAD (mmol/kg FW)	0.04 ± 0.01	0.79 ± 0.19	0.15 ± 0.14	0.97 ± 0.22
Range (% total)	2–5	78–83	13–18	–
Median ± MAD (% total)	4 ± 1	82 ± 1	15 ± 1	–

### Alfalfa Caffeic Acid *O*-Methyltransferase (COMT) Is Incapable of Direct Methylation of Caffeoyl-Malate

Lack of accumulation of caffeoyl-malate and accumulation of feruloyl-malate in leaves of alfalfa expressing red clover HMT suggested two non-mutually exclusive mechanisms whereby feruloyl-malate might be produced. The first would be that feruloyl-malate is produced directly from caffeoyl-malate by the action of caffeic acid *O*-methyltransferase (COMT). Alternatively, caffeoyl or caffeoyl-CoA moieties might be converted to feruloyl moieties by the action of either COMT or caffeoyl-CoA *O*-methyltransferase (CCOMT) and the resulting feruloyl moieties converted to feruloyl-malate by HMT (following ligation to CoA by 4-coumarate CoA ligase for free ferulic acid) (Figure 1).

**FIGURE 4:**
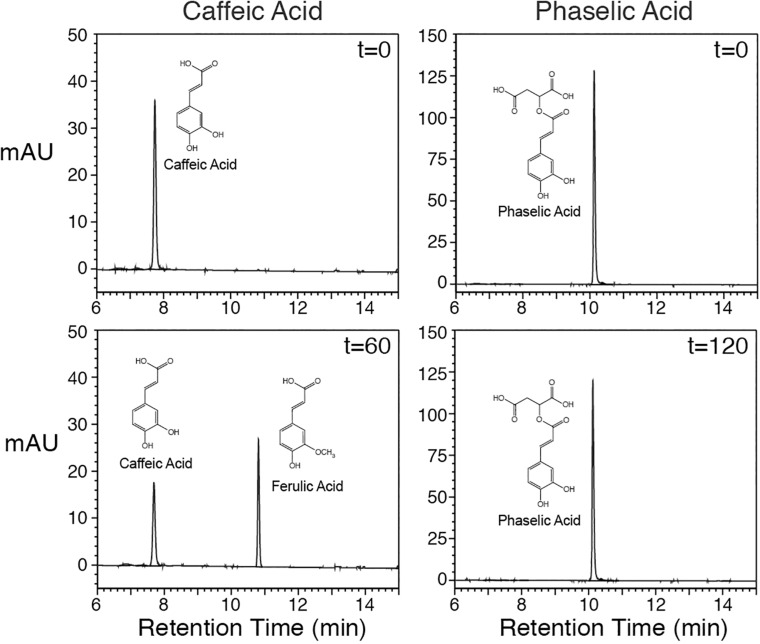
Alfalfa COMT protein, produced in *E. coli*, was incubated with caffeic acid **(left panels)** or caffeoyl-malate **(right panels)** for up to 120 min as indicated, and reactions were resolved by reverse phase LC with PDA detection (250–500 nm). Reactants and products are labeled. No methylation product was detected when caffeoyl-malate was used as substrate.

In order to assess whether COMT is capable of directly converting caffeoyl-malate to feruloyl-malate, an alfalfa cDNA encoding COMT was cloned for expression in *E. coli*. The cDNA (Genbank GU066087) encodes a protein that has a single amino acid substitution (asparagine for isoleucine at position 88) relative to a previously reported COMT cDNA (M63853; Gowri et al., 1991), but the substitution likely represents natural variation, as other *M. sativa* sequences in Genbank have this substitution. The cDNA was expressed in *E. coli*, and the resulting COMT protein’s *in vitro* enzyme activities were assessed. Expression of the alfalfa COMT cDNA in *E. coli* resulted in active enzyme that was able to methylate caffeic acid to ferulic acid in the presence of *S*-adenosyl methionine (Figure 4, left panels at 0 and 60 min) as previously demonstrated by others (Gowri et al., 1991). Methylation was dependent on the COMT protein as no reaction was seen with extract of *E. coli* transformed with the pET vector only (data not shown). Incubation of caffeoyl-malate with the *E. coli* expressed COMT and *S*-adenosyl methionine did not result in conversion of caffeoyl-malate to the corresponding feruloyl ester (Figure 4, right panels at 0 and 120 min). This finding indicates that alfalfa COMT is incapable of directly methylating caffeoyl-malate to feruloyl-malate. Thus, it seems likely feruloyl-malate is formed in alfalfa *in vivo* by HMT utilization of feruloyl-CoA as donor substrate, although we cannot absolutely rule out the existence of an uncharacterized *O*-methyltransferase activity capable of carrying out this transformation *in vivo*.

### Downregulation of Endogenous CCOMT Enhances Accumulation of Caffeoyl-Malate in Alfalfa Plants Expressing Red Clover HMT

Given the above results, we hypothesized that downregulation of endogenous alfalfa CCOMT might increase accumulation of caffeoyl-malate at the expense of feruloyl-malate without necessarily affecting accumulation of *p*-coumaroyl-malate or total hydroxycinnamoyl-malate levels. To test this hypothesis, two alfalfa plants already transformed with red clover HMT (#17 and #56) and showing substantial foliar accumulation of *p*-coumaroyl- and feruloyl-malate (on the order of 1 mmol/kg FW) were supertransformed with a CCOMT RNAi construct. The empty vector used for the RNAi construct was supertransformed as a negative control. Numerous (5–19) independent transformation events, confirmed by PCR for the presence of transgenes, for each of the four combinations (two different backgrounds transformed with RNAi or vector control) were recovered. Phenolics present in leaves were identified and quantified by LC. An LC trace for one of the HMT CCOMT RNAi plants (Figure 2) shows a dramatic increase in caffeoyl-malate accumulation relative to the parental plant when the CCOMT RNAi is present. Substantial increases in caffeoyl-malate accumulation relative to the parental plants were seen for about half (10 of 21) of the RNAi supertransformed plants, while none of the plants supertransformed with vector (control) were obviously different from the parental plants with respect to caffeoyl-malate accumulation (see Supplementary Data). None of the plants (RNAi or vector control) had any obvious morphological or developmental phenotype compared to wild type alfalfa plants.

**FIGURE 5:**
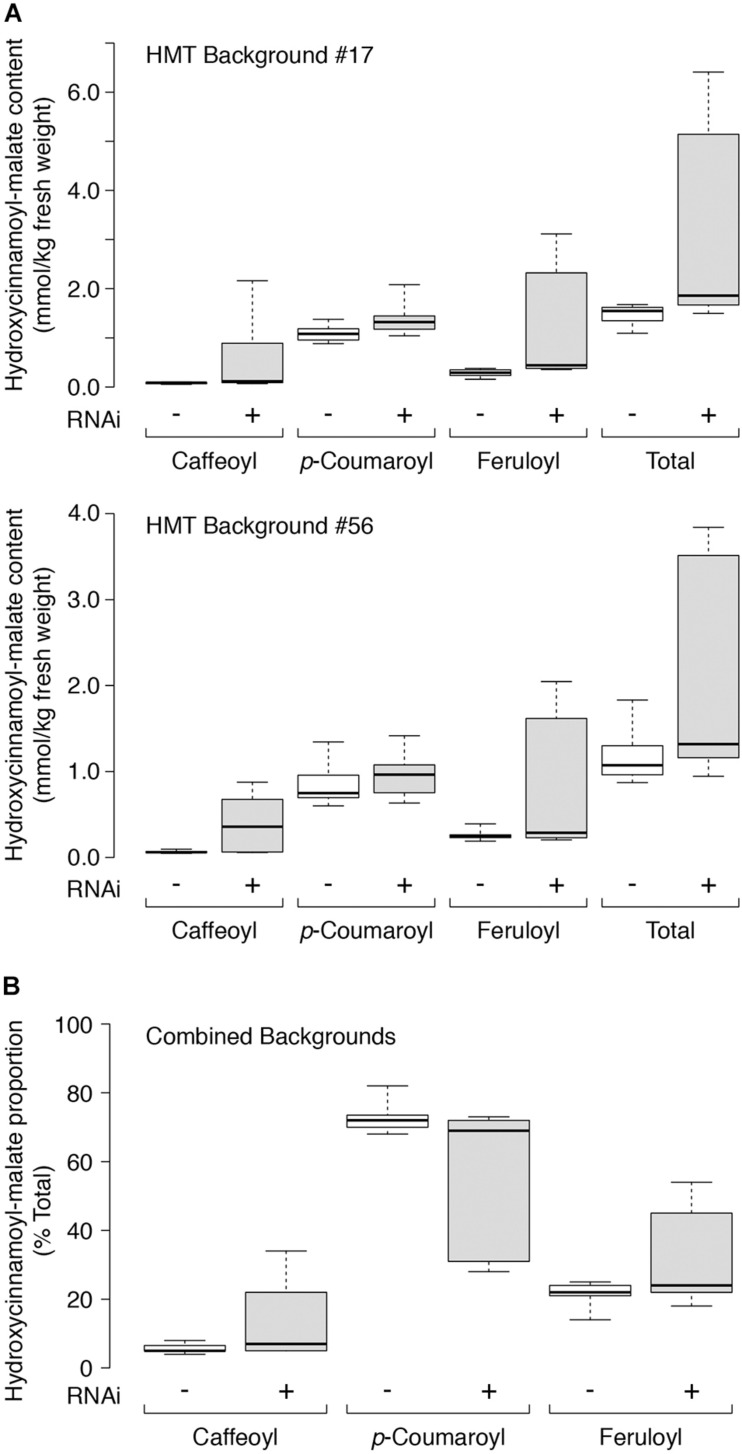
**(A)** Hydroxycinnamoyl-malate content in leaves of HMT alfalfa plants retransformed with vector (negative control, –, white boxes) or CCOMT RNAi (+, gray boxes). Two different HMT alfalfa backgrounds (#17 and #56) were used as indicated with a minimum of five independent transformants in each group. **(B)** Proportion of individual hydroxycinnamoyl-malate esters as percent of total for leaves in both backgrounds with 24 and 21 independent transformants analyzed for the vector and RNAi groups, respectively. Labels are as in A above. For all panels, center lines show the medians, box limits indicate the 25th and 75th percentiles as determined by R software, whiskers extend to minimum and maximum values.

Quantitative accumulation data for all plants are represented in box and whiskers plots (Figure 5A) with summary statistics in Table 3 (see also Supplementary Data). Because the two HMT backgrounds used for the supertransformation experiment were derived from two independent HMT transformation events, they were analyzed separately with respect to hydroxycinnamoyl-malate ester accumulation. Even though about half of the RNAi plants had no obvious phenotype with respect to caffeoyl-malate accumulation, as a whole they had significantly higher levels of caffeoyl-malate (*P* < 0.05) and total hydroxycinnamoyl-malate esters (*P* < 0.05), regardless of background. Although in most cases median accumulation for these compounds was not substantially higher for the RNAi plants, this was largely driven by the relatively high proportion of plants included in the analysis which lacked an obvious caffeoyl-malate phenotype. That the RNAi construct is impacting caffeoyl- and total hydroxycinnamoyl-malate accumulation is more apparent from the box and whisker representations of the data (Figure 5A), where the top two quartiles for the RNAi plants show little overlap with the control plants. *p*-Coumaroyl-malate accumulation in the RNAi plants, as a whole, was not significantly different from the control plants (*P* = 0.075 and *P* = 0.123 for the #17 and #56 backgrounds, respectively) although there may be a trend toward an increase in the RNAi plants. Surprisingly, despite downregulation of CCOMT, feruloyl-malate levels seemed to increase in the RNAi plants [significant in #17 background (*P* = 0.003) and trending in the #56 background (*P* = 0.085)], which is also well-reflected in the box and whiskers representation of the data, where the top two quartiles for the RNAi plants show little overlap with the control plants.

**TABLE 3:** Accumulation of hydroxycinnamoyl esters in leaves of plants transformed with HMT and CCOMT RNAi or vector control.

Background	RNAi	n	*trans*-Hydroxycinnamoyl-malate content (mmol/kg fresh weight)							
			
			Caffeoyl		*p*-Coumaroyl		Feruloyl		Total	
						
			Range	Median^a^	Range	Median	Range	Median	Range	Median
17	−	5	0.05–0.10	0.09 ± 0.01	0.88–1.38	1.08 ± 0.12	0.16–0.38	0.29 ± 0.05	1.09–1.68	1.55 ± 0.13
	+	10	0.07–2.16	0.11 ± 0.03	1.04–2.08	1.32 ± 0.14	0.35–3.12	0.44 ± 0.07	1.50–6.41	1.86 ± 0.25
*P*-value^b^				0.038^c^		0.075		0.003		0.028
56	−	19	0.05–0.10	0.06 ± 0.01	0.60–1.34	0.75 ± 0.07	0.19–0.39	0.25 ± 0.13	0.87–1.83	1.07 ± 0.13
	+	11	0.06–0.88	0.35 ± 0.29	0.63–1.42	0.96 ± 0.15	0.20–3.84	1.32 ± 0.38	0.94–3.84	1.32 ± 0.38
*P*-value				0.004^c^		0.123		0.085		0.021

*^*a*^±MAD. ^*b*^P-value based on Mann-Whitney test. ^*c*^One-tailed P-value based on directional hypothesis that level of caffeoyl esters would increase.*

Although some differences in accumulation of both the total hydroxycinnamoyl-malate esters as well as the individual derivatives were only marginally significant (*P* just under 0.05), trending (0.05 < *P* < 0.10), or not significant (*P* > 0.10) by the Mann-Whitney analysis, as discussed above, this may be due to including all plants containing the transgenes of interest in the analysis. It is possible that high level suppression of CCOMT expression might interfere with recovery of transgenic plants in our system, which could lead to recovery of a higher proportion of plants with low level or no silencing. Thus, it might not be surprising that for plants supertransformed with the RNAi construct, nearly half showed no discernable increase in hydroxycinnamoyl-malate esters, while some plants had dramatic increases in hydroxycinnamoyl-malate esters (Figure 5A).

Although downregulation of CCOMT was expected to increase caffeoyl-malate accumulation, it is seemingly paradoxical that not only did feruloyl-malate accumulation not decrease, it appears to have increased. It could be that downregulation of CCOMT increased the pool size of caffeoyl-CoA, leading to the expected increase in caffeoyl-malate accumulation. An alternative pathway to feruloyl moiety biosynthesis, for example by COMT either on its own, since COMT is capable of methylating caffeoyl-CoA (Inoue et al., 1998), or in conjunction with esterases that might liberate free caffeoyl moieties from caffeoyl-shikimate (Ha et al., 2016) or caffeoyl-CoA, then subsequent religation of the newly formed feruloyl moiety to CoA by 4-coumarate:CoA ligase, would allow feruloyl-malate to be formed by HMT (Figure 1). Whether an altered caffeoyl-CoA pool size would allow such a pathway to work efficiently, and whether some sort of feedback mechanism might allow flow of metabolites in this way is unclear. We are currently planning detailed transcriptomic and metabolomic studies of HMT/CCOMT RNAi plants that should provide additional insight into this.

For each independent line, we also determined the proportion of each hydroxycinnamoyl-malate ester. Since we did not expect HMT background (#17 or #56) to impact the relative proportion of the various hydroxycinnamoyl esters, we combined data from both backgrounds and compared the presence or absence of the RNAi silencing construct using Mann-Whitney analysis. A box and whiskers representation of the data is presented in Figure 5B and summary statistics are presented in Table 4 (see also Supplementary Data). The proportion of caffeoyl-malate increased in the group of plants supertransformed with the RNAi silencing construct (*P* = 0.004, one-tailed), as did the proportion of feruloyl-malate (*P* = 0.007), while the proportion of *p*-coumaroyl-malate decreased (*P* = 0.003). As with the analysis of absolute amounts of hydroxycinnamoyl-malate esters, because many of the RNAi plants included in the analysis did not have a phenotype, these differences were not always apparent from median values. However, as can be seen in Figure 5B, for RNAi plants the proportion of caffeoyl- and feruloyl-malate for plants above the median values are substantially higher than those of nearly all plants for the vector control, whereas for RNAi plants the proportion of *p*-coumaroyl-malate for plants below the median value are substantially lower than those of nearly all the vector control plants. Further, increases in the proportion of caffeoyl-malate and feruloyl-malate were correlated with each other (*R*^2^ = 0.288, *P* = 0.012) and with a decrease in the proportion of *p*-coumaroyl-malate (*R*^2^ = 0.701 and 0.829 for caffeoyl- and feruloyl-malate, respectively, *p* < 0.0001 for both; see also Supplementary Data). The highest level of caffeoyl-malate accumulation measured for leaves of these plants was 2.16 mmol/kg FW, which constituted 34% of hydroxycinnamoyl-malate esters accumulating.

**TABLE 4:** Proportion of various hydroxycinnamoyl esters in leaves of plants transformed with HMT and CCOMT RNAi or vector control.

Background	RNAi	n	*trans*-Hydroxycinnamoyl-malate content (percent of total)					
			
			Caffeoyl		*p*-Coumaroyl		Feruloyl	
					
			Range	Median^a^	Range	Median	Range	Median
Combined	−	24	4–8	5 ± 0	68–82	72 ± 2	14–25	22 ± 1
	+	21	5–34	7 ± 2	28–73	69 ± 4	18–54	24 ± 2
*P*-value^b^				0.004^c^		0.003		0.007

*^*a*^±MAD. ^*b*^P-value based on Mann-Whitney test. ^*c*^One-tailed P-value based on directional hypothesis that proportion of caffeoyl esters would increase.*

### Accumulation of Caffeoyl-Malate Is Enhanced in Stems Relative to Leaves for HMT/CCOMT RNAi Alfalfa

**FIGURE 6:**
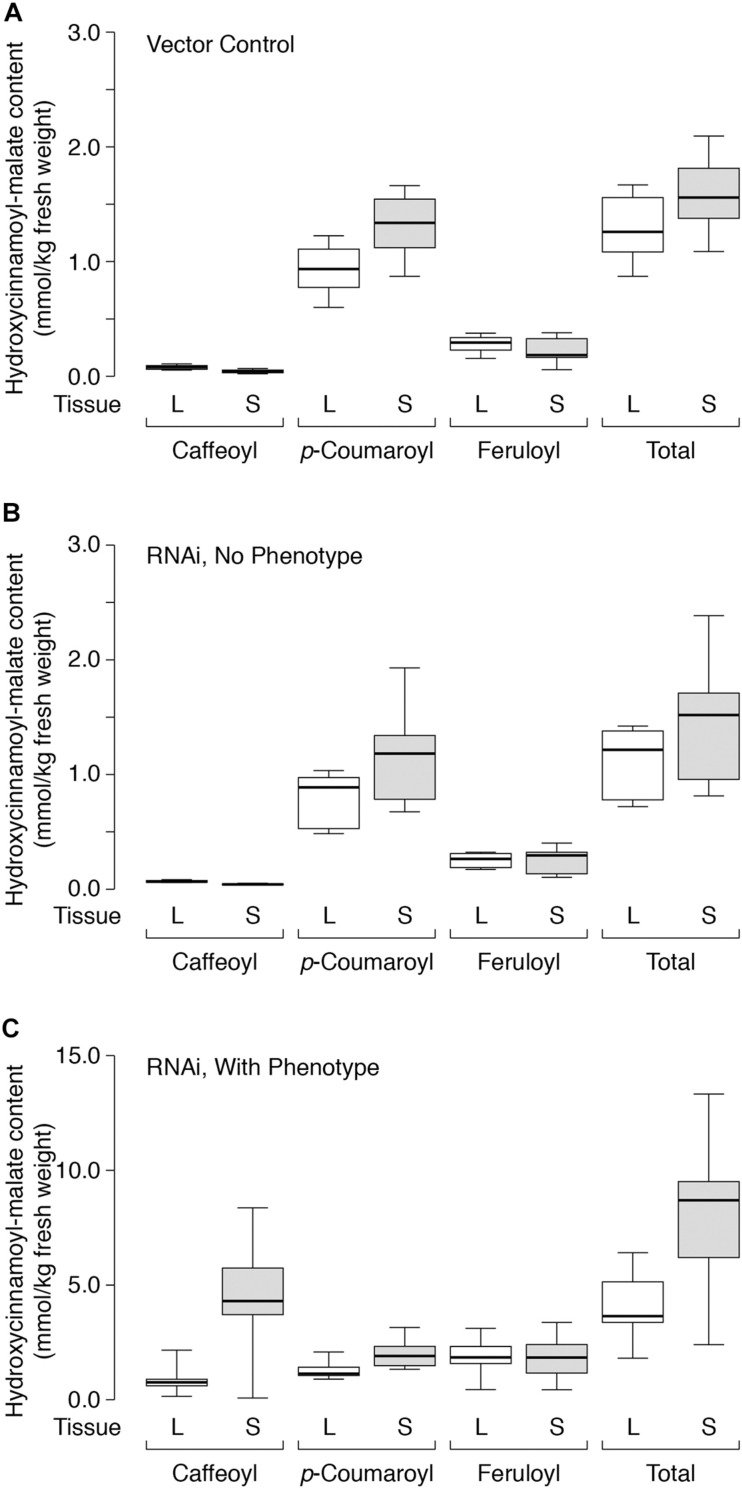
Hydroxycinnamoyl-malate accumulation in leaves (L, white boxes) versus stems (S, gray boxes) of HMT-expressing alfalfa **(A)** retransformed with vector only (eight independent transformants), **(B)** retransformed with the CCOMT RNAi construct but not showing the caffeoyl-malate phenotype (six independent transformants), or **(C)** retransformed with the CCOMT RNAi construct and showing the caffeoyl-malate phenotype (10 independent transformatns). Center lines show the medians, box limits indicate the 25th and 75th percentiles as determined by R software, whiskers extend to minimum and maximum values.

Many of the enzymes involved in phenylpropanoid biosynthesis in general (phenylalanine ammonia lyase, cinnamic acid 4-hydroxylase, 4-comaric acid CoA ligase) (see (Dixon et al., 2001) and references therein) and caffeoyl moiety biosynthesis specifically [hydroxycinnamoyl-CoA:shikimate transferase (HST), C3′H] (see for example Sullivan, 2009; Sullivan and Zarnowski, 2010) are highly expressed in stems, presumably to provide monolignols for lignin biosynthesis. In *Medicago truncatula*, a model legume closely related to alfalfa, expression of HST and C3′H are three to fourfold higher in lignifying stems compared to leaves based on data from the *Medicago truncatula* Gene Expression Atlas^5^ (see Supplementary Figure 1). Consequently, we hypothesized that hydroxycinnamoyl-malate ester accumulation, particularly caffeoyl-malate accumulation, might be higher in stems than in leaves for alfalfa expressing HMT and/or CCOMT RNAi. For representative plants analyzed above, stem hydroxycinnamoyl-malate content was also analyzed and compared to that of leaves, and differences were evaluated using the Wilcoxon sign-rank test. The plants examined were placed into three categories without regard to the HMT background: vector control plants lacking the CCOMT RNAi transgene, plants transformed with the RNAi transgene but not showing a phenotype (defined as having foliar caffeoyl-malate accumulation ≤ 0.10 mmol/kg FW, the highest measured level for vector control plants, “RNAi, no phenotype”), and plants transformed with the RNAi transgene showing a phenotype (defined as having foliar caffeoyl-malate accumulation > 0.10 mmol/kg FW, “RNAi, with phenotype”). Results of the analysis are shown in Table 5 and Figure 6.

**TABLE 5:** Accumulation of hydroxycinnamoyl esters in leaves (L) and stems (S) of plants transformed with HMT and CCOMT RNAi (without and with the caffeoyl-malate accumulation phenotype) or vector control.

Group	L/S	n	*trans*-Hydroxycinnamoyl-malate content (mmol/kg fresh weight)							
			
			Caffeoyl		*p*-Coumaroyl		Feruloyl		Total	
						
			Range	Median^a^	Range	Median	Range	Median	Range	Median
Vector	L	8	0.05–0.11	0.08 ± 0.02	0.60–1.23	0.93 ± 0.17	0.16–0.38	0.29 ± 0.05	0.87–1.67	1.26 ± 0.24
	S		0.02–0.07	0.05 ± 0.01	0.87–1.66	1.34 ± 0.21	0.06–0.38	0.18 ± 0.07	1.09–2.09	1.56 ± 0.21
*P*-value^b^				0.008		0.004^c^		0.461		0.008^c^
RNAi, no phenotype	L	6	0.06–0.08	0.07 ± 0.01	0.48–1.04	0.89 ± 0.12	0.17–0.32	0.26 ± 0.05	0.72–1.42	1.22 ± 0.18
	S		0.04–0.05	0.04 ± 0.01	0.67–1.93	1.18 ± 0.28	0.10–0.40	0.29 ± 0.07	0.81–2.38	1.52 ± 0.38
*P*-value				0.003		0.002^c^		0.843		0.002^c^
RNAi, with phenotype	L	10	0.14–2.16	0.76 ± 0.14	0.90–2.08	1.14 ± 0.09	0.44–3.11	1.84 ± 0.38	1.82–6.41	3.65 ± 0.70
	S		0.07–8.37	4.30 ± 1.20	1.33–3.15	1.91 ± 0.42	0.44–3.37	1.84 ± 0.62	2.40–13.32	8.69 ± 2.38
*P*-value				0.004^c^		0.001^c^		0.688		0.001^c^

*^*a*^±MAD. ^*b*^P-value from Wilcoxon signed-rank sign test. Two-tailed P-values are shown unless otherwise noted. ^*c*^Observation followed the directional hypothesis (higher hydroxycinnamoyl-malate ester content in stems versus leaves), so one-tailed P-value is shown.*

Not surprisingly, the vector control plants and the RNAi plants lacking phenotype showed similar accumulation patterns for the various hydroxycinnamoyl-malate esters in leaves versus stems (Figure 6, Table 5, and Supplementary Data). For these groups, caffeoyl-malate levels in stems were slightly lower relative to leaves [*P* = 0.008 and 0.003 (two-tailed)] rather than higher as might have been expected, and feruloyl-malate accumulation was not significantly different between leaves and stems (*P* = 0.46 and 0.84 [two-tailed]). In contrast, *p*-coumaroyl-malate content was significantly higher in stems relative to leaves in both groups [*P* < 0.002 (one-tailed)], although the magnitude of the difference was less than twofold. The higher level of *p*-coumaroyl-malate content in stems appears to account for a higher level of total hydroxycinnamoyl-malate accumulation (*P* < 0.008).

For the RNAi plants with phenotype (increased accumulation of caffeoyl-malate in leaves), like the vector control and no phenotype plants, feruloyl-malate accumulation was not significantly different between leaves and stems (*P* = 0.69) and *p*-coumaroyl-malate accumulation was significantly higher in stems compared to leaves [*P* = 0.001 (one-tailed)]. In contrast, median accumulation of caffeoyl-malate was fivefold higher in stems relative to leaves [4.30 ± 1.20 versus 0.76 ± 0.14 mmol/kg fresh weight (median ± MAD), *P* = 0.004 (one-tailed)]. The higher levels of both *p*-coumaroyl-malate and caffeoyl-malate account for significantly higher levels of total hydroxycinnamoyl-malate in stems compared to leaves for the RNAi plants [8.69 ± 2.38 versus 3.65 ± 0.70 mmol/kg fresh weight (median ± MAD), *P* = 0.002 (one-tailed)]. The highest measured accumulation of caffeoyl-malate was 8.37 mmol/kg fresh weight, accounting for 63% of the hydroxycinnamoyl-malate present in stems in this plant. Although this plant had both the highest absolute amount as well as proportion of caffeoyl-malate, most (except for one outlier) had a high proportion of caffeoyl-malate (52 ± 7%, median ± MAD), and lesser proportions of *p*-coumaroyl- and feruloyl-malate (24 ± 1 and 20 ± 4%, respectively, median ± MAD) (see also Supplementary Data). For stems, levels of hydroxycinnamoyl-malate esters in general, and caffeoyl-malate in particular, are comparable to those found in leaves of red clover, indicating a huge potential in reshaping specialized metabolism in alfalfa. It seems likely that the normally high levels of phenylpropanoid biosynthetic enzymes in stems are supporting high levels of caffeoyl-malate biosynthesis and accumulation. Using additional transgenes to increase levels of some of these enzymes in leaves, for example hydroxycinnamoyl-Co:A: shikimate hydroxycinnamoyl transferase and C3′H to increase levels of caffeic acid, might similarly lead to increased levels of foliar caffeoyl-malate accumulation. Aside from increased levels of hydroxycinnamoyl-malate in leaves and stems of the transgenic plants, no obvious phenotypes were observed for greenhouse grown plants. It is not clear whether the redirection of specialized metabolism in these plants would impact agronomic performance either negatively (for example, by diverting resources or altering lignin structure) or positively (for example, enhancing defense against biotic and abiotic stresses). We anticipate evaluating field performance of these plants alone and in combination with a PPO transgene in future experiments to evaluate the complete PPO system (see below).

### Extent of CCOMT Downregulation in Alfalfa Accumulating Caffeoyl-Malate

To evaluate the extent to which CCOMT was downregulated in alfalfa plants accumulating caffeoyl-malate, CCOMT expression was examined by real-time PCR in five independent transformants from the “RNAi, with phenotype” group and compared with that of four independent transformants from the vector control group. The CCOMT primer pair amplified a segment of the CCOMT coding region outside of the segment used in the RNAi silencing construct to avoid interference from the silencing RNA. Results of this analysis are shown in Table 6. As expected, alfalfa in the “RNAi, with phenotype” group had a significant decrease (approximately 50-fold less, on average) in CCOMT mRNA relative to vector control plants (*P* < 0.001). Unfortunately, only a single plant from the “RNAi, no phenotype” group was analyzed, making statistical analysis not possible. However, this single plant had a CCOMT mRNA level indistinguishable from those measured for vector control plants, consistent with the idea that alfalfa plants transformed with the CCOMT RNAi construct but which fail to accumulate higher levels of caffeoyl-malate are not sufficiently downregulated in CCOMT to exhibit the caffeoyl-malate accumulation phenotype. Because the “RNAi, with phenotype” group had enhanced levels of feruloyl-malate accumulation despite CCOMT downregulation, we also examined COMT mRNA levels. No significant differences (*P* > 0.05) in COMT expression were detected between vector control and “RNAi, with phenotype” plants. These results indicate that COMT expression is not enhanced in response to CCOMT downregulation. Thus, wild type levels of COMT expression are sufficient for the enhanced accumulation of feruloyl-malate in CCOMT downregulated plants, and the enhanced accumulation may be due to changes in precursor pool sizes.

**TABLE 6:** Real-time PCR analysis of CCOMT and COMT expression.

Group	n	−Log_2_ Fold Change (ΔΔC_T_, relative to vector)	
		
		CCOMT	COMT
Vector	4	0.00 ± 0.38*	0.00 ± 0.11
RNAi, with phenotype	5	5.26 ± 0.18*	−0.31 ± 0.10
RNAi, no phenotype	1	−0.01	−0.28

*Marked entries within a column are significantly different at P < 0.001. All other differences are not significant at P > 0.05. “RNA, no phenotype” was not included in the statistical analysis.

### PPO-Mediated Proteolytic Inhibition Is Correlated With Caffeoyl-Malate Content in Transgenic Alfalfa

**FIGURE 7:**
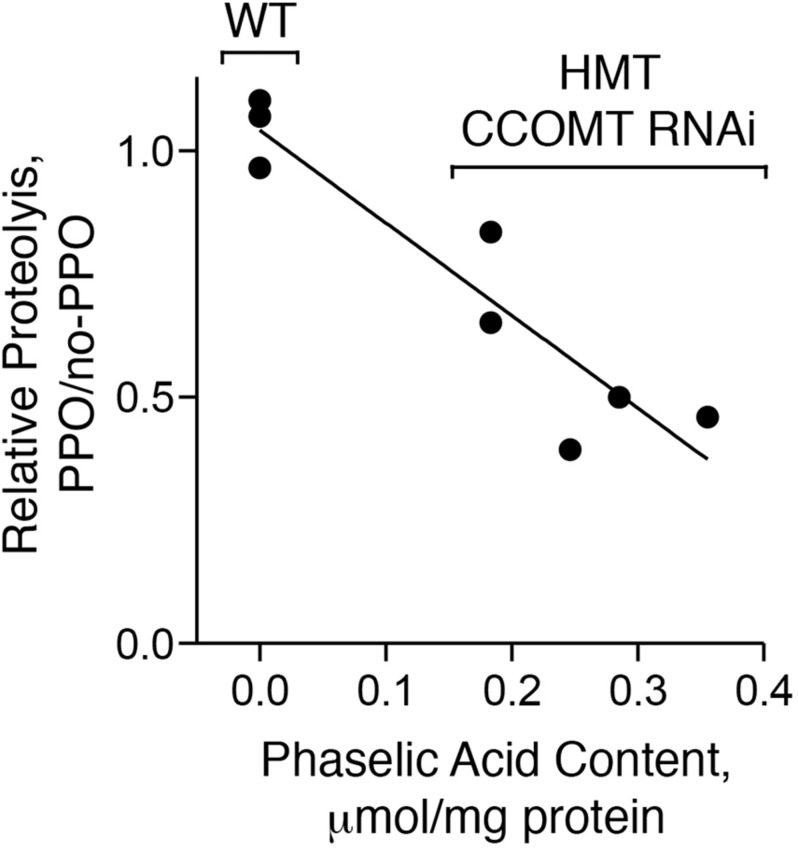
PPO-dependent proteolytic inhibition is correlated with caffeoyl-malate content. Extracts of five transgenic (HMT and CCOMT RNAi) and three wild type plants (WT) were analyzed for caffeoyl-malate content and PPO-dependent proteolytic inhibition. Correlation of PPO-dependent proteolytic inhibition with caffeoyl-malate content was assessed by regression analysis. For the wild type plants, only the median value of the three plants assayed was included in the regression analysis.

We wished to test whether the alfalfa expressing red clover HMT and CCOMT RNAi produced sufficient caffeoyl-malate to inhibit protein breakdown by endogenous proteases in a PPO-dependent manner. Alfalfa leaves and stems do not express an endogenous PPO gene (Sullivan et al., 2004, 2008; Sullivan and Hatfield, 2006), but alfalfa transformed with red clover PPO has been produced (Sullivan et al., 2004). The PPO trait and the caffeoyl-malate trait are currently not both present in the same plant, but we were able to examine PPO-dependent proteolytic inhibition by an *in vitro* reconstruction of the system. Extracts were prepared from stems and leaves of five caffeoyl-malate-producing transgenic alfalfa plants and three wild type, non-transformed control plants. Caffeoyl-malate and protein content of the extracts were measured. Leaf extracts from alfalfa plants expressing PPO served as the source of PPO while leaf extracts from non-transformed plants served as a negative (no-PPO) control. Reactions containing 90% (v/v) of the leaf and stem (caffeoyl-malate-containing) and 10% (v/v) of the PPO or no-PPO leaf extract were incubated and proteolysis (as amino acid release) was measured. PPO-dependent proteolytic inhibition in the caffeoyl-malate-containing extracts was assessed by comparing amino acid release in the presence versus absence of PPO (Figure 7).

Wild type control plants (not expressing HMT or CCOMT RNAi) did not contain caffeoyl-malate, while the transgenic plants had caffeoyl-malate concentrations that ranged from approximately 0.2–0.4 μmol per mg protein. For wild type plants (no caffeoyl-malate) amino acid release over the 24 h time period ranged from 1.2 to 1.7 μmol per mg of protein for the individual extracts and was unchanged by the presence of PPO (thus ratio of proteolysis with PPO versus no-PPO ranged from 0.97 to 1.10). This was expected as both PPO and a PPO substrate such as caffeoyl-malate are required for proteolytic inhibition (Sullivan and Hatfield, 2006). For the caffeoyl-malate-producing plants, amino acid release in the absence of PPO ranged from about 1.5–2 mmol per mg of protein. However, in contrast to the wild type plants, it was reduced in the presence of PPO by 17–60% and PPO-dependent reductions in proteolysis were highly correlated with caffeoyl-malate content of the plant extract (*r*^2^ = 0.81, *P* = 0.004).

The extent of proteolytic inhibition seen for the caffeoyl-malate-containing alfalfa extracts (∼50%) is similar to that seen in previous experiments using purified caffeoyl-malate (Sullivan and Zeller, 2012) and might be expected to have substantial impacts on proteolytic losses during ensiling and N-utilization in ruminant animals. It must be kept in mind, however, that the non-uniform distribution of caffeoyl-malate in the transgenic alfalfa (more in stems than in leaves) could result in less proteolytic inhibition during ensiling than is seen in these extract experiments. Nonetheless, previous experiments utilizing PPO-expressing transgenic alfalfa treated with exogenously applied PPO substrate, showed substantial reductions in proteolysis in mini ensiling experiments when the tissue was well-macerated (Sullivan and Hatfield, 2006).

## Conclusion

Here we’ve shown that transgenic expression of red clover HMT in alfalfa results in accumulation of hydroxycinnamoyl-malate derivatives. When endogenous CCOMT was also downregulated, caffeoyl-malate accumulated to substantial levels (up to about 2 and 8 mmol/kg FW in leaves and stems, respectively). The levels of caffeoyl-malate produced in these plants is sufficient to reduce proteolysis in the presence of PPO in *in vitro* experiments. The next step will be to cross PPO-expressing alfalfa plants with caffeoyl-malate-producing alfalfa plants to generate experimental populations of alfalfa (PPO-expressing, caffeoyl-malate-producing, PPO-expressing and caffeoyl-malate-producing, and wild type). These populations should allow us to better assess how well this level of PPO substrate production and distribution within the plant (more in stems than in leaves) will perform during ensiling, and whether ensiling conditions (e.g., extent of maceration) will need to be optimized to take advantage of the PPO system. These materials should also allow us to begin examining how the PPO system might impact N-utilization and animal performance first via *in vitro* experiments, but ultimately in small ruminant feeding studies. We should also be able to evaluate other impacts of these traits on field performance and resistance to biotic and abiotic stress. Lignin content and composition will also be evaluated, as it could be altered in this system due to the CCOMT downregulation, the diversion of metabolites away from monolignols by HMT expression, or both. Altered lignin composition could result in higher fiber digestibility in ruminant production systems, thus delivering additional value.

## Data Availability Statement

The datasets presented in this study can be found in the Supplementary Material.

## Author Contributions

MS, HG, and JV contributed to conception and design of the study. MS and HG carried out the experiments related to the transgenic alfalfa. JV carried out the COMT experiment. MS carried out data analysis and wrote the first draft of the manuscript. All authors contributed to manuscript revision, read, and approved the submitted version.

## Conflict of Interest

This work was supported in part by Forage Genetics International. Beyond this relationship, the authors declare that the research was conducted in the absence of any commercial or financial relationships that could be construed as a potential conflict of interest.

## Footnotes

1 www.ncbi.nlm.nih.gov

2 http://shiny.chemgrid.org/boxplotr

3 http://www.statskingdom.com/170median_mann_whitney.html

4 https://www.graphpad.com/quickcalcs/binomial1/

5 https://mtgea.noble.org/v3/
