# Supplementary material for: Engineering Alfalfa to Produce 2-O-Caffeoyl-L-Malate (Phaselic Acid) for Preventing Post-harvest Protein Loss via Oxidation by Polyphenol Oxidase
Source: Front Plant Sci. 2021 Jan 13;11:610399. doi: 10.3389/fpls.2020.610399 (PMC7838361; doi:10.3389/fpls.2020.610399)
Supplement: Supplementary file 1 [file Data_Sheet_1.PDF]

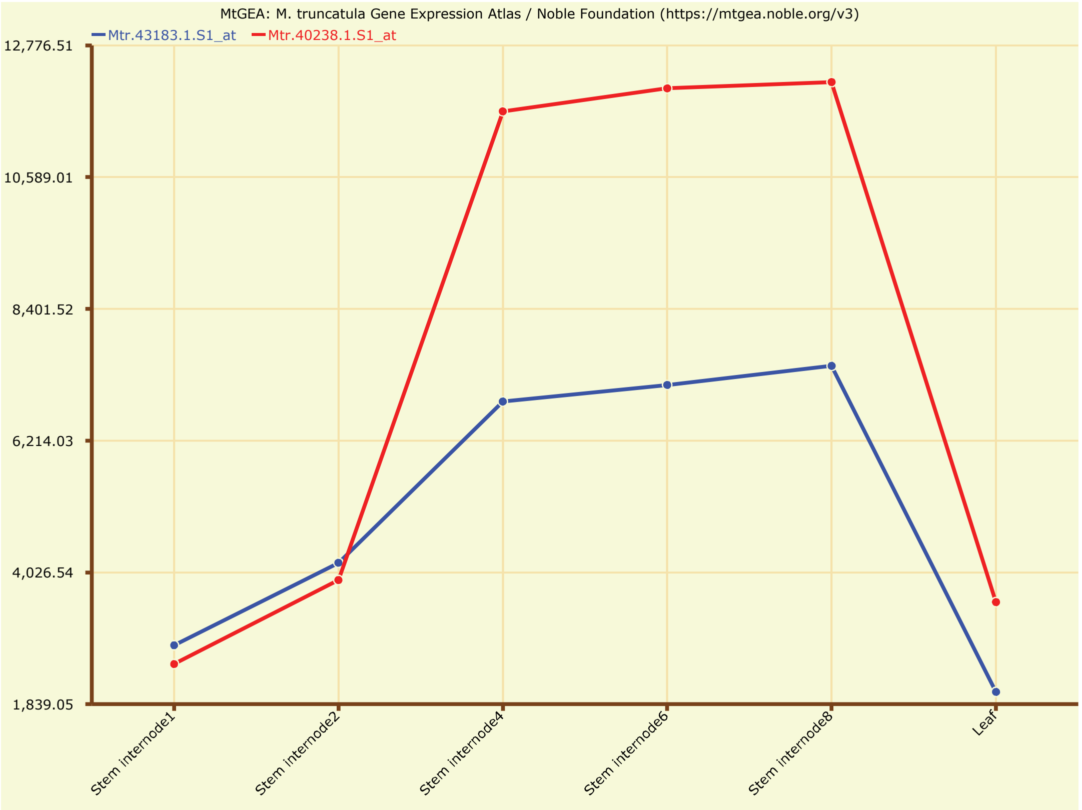

Supplemental Figure. Transcript levels in *Medicago truncatula* stem internodes or leavves for HST (Mtr.40238.1.S1_at, red) or C3'H (Mtr.43183.1.S1_at, blue) based on Medicago truncatula Gene Expression Atlas microarray data available at mtgea.noble.org/v3. Probes were identified by BLAST search of the Medicago Affy Target Sequences database using red clover HST or C3'H sequences (Genbank FJ151489 and GQ919201, respectively) as queries. The visualization was created using the atlas's online MTV tool.
